# Quality control and biophysical characterisation data of *VanS*_*A*_

**DOI:** 10.1016/j.dib.2017.07.012

**Published:** 2017-07-13

**Authors:** C.S. Hughes, E. Longo, M.K. Phillips-Jones, R. Hussain

**Affiliations:** a Diamond Light Source, Harwell Research & Innovation Campus, Chilton, Didcot OX11 0DE, United Kingdom; b Membranes, Membrane Proteins & Peptides Research Group, School of Pharmacy & Biomedical Sciences, University of Central Lancashire, Preston PR1 2HE, United Kingdom

## Abstract

This data article presents the results from quality control experiments including N-terminal sequencing, SEC-MALS and Mass Spectrometry for purified VanS_A_ used in experiments described in (Hughes et al., 2017) [1]; in addition to ligand interaction measurements and thermal melting curves of VanS_A_ in the presence of screened ligands from circular dichroism measurements as well as UV–vis absorbance spectra for the binding interaction of VanS_A_ in the presence of screened ligands.

## **Specifications Table**

**Table:** 

Subject area	Biology
More specific subject area	Antibiotic Resistance
Type of data	Image of PVDF membrane and N-terminal sequence results; SEC-MALS spectrum, buffer baseline subtracted; Mass Spectrometry trypsin-digest fragments table and spectrum; thermal denaturation melt profile at 225 nm obtained from CD spectra. UV–vis absorbance spectra of VanS_A_ in the presence of screened ligands.
How data was acquired	Western blot, SEC-MALS, trypsin-digest mass spectrometry, CD, UV–vis absorbance
Data format	N-terminal sequencing by Edman degradation; SEC-MALS analysed by buffer baseline subtraction; mass spectrometry; Raw MS data were processed by MaxQuant (version 1.5.0.35i) for peak detection and quantification and against a custom database using the Andromeda search engine; CD spectra analysed using CDApps [2]; thermal denaturation profiled plotted in OriginPro and fitted with Gibbs-Helmholtz equation derived from Boltzmann distribution [3], [4].
Experimental factors	Detergent removal and trypsin digest before mass spectrometry, incubation with ligand for 30 minutes priot to data collection for CD.
Experimental features	Quality control of protein preparations using N-terminal sequencing, SEC-MALS and mass spectrometry prior to CD thermal melt experiments (20–95 °C in 5 °C increments).
Data source location	
Data accessibility	Data in article

## **Value of the data**

• Data is indicative of quality control measurements for protein preparations for accompanying experiments detailed in research article.
• Amino acid sequencing for the recombinant expression of His_6_-tagged VanS_A_.
• Thermal melt profiles @ 225 nm for data summarised in Table 1 in Research article [1].
• Binding interaction further monitored by UV–vis absorbance.

## 1 Data

**Fig. 1:**
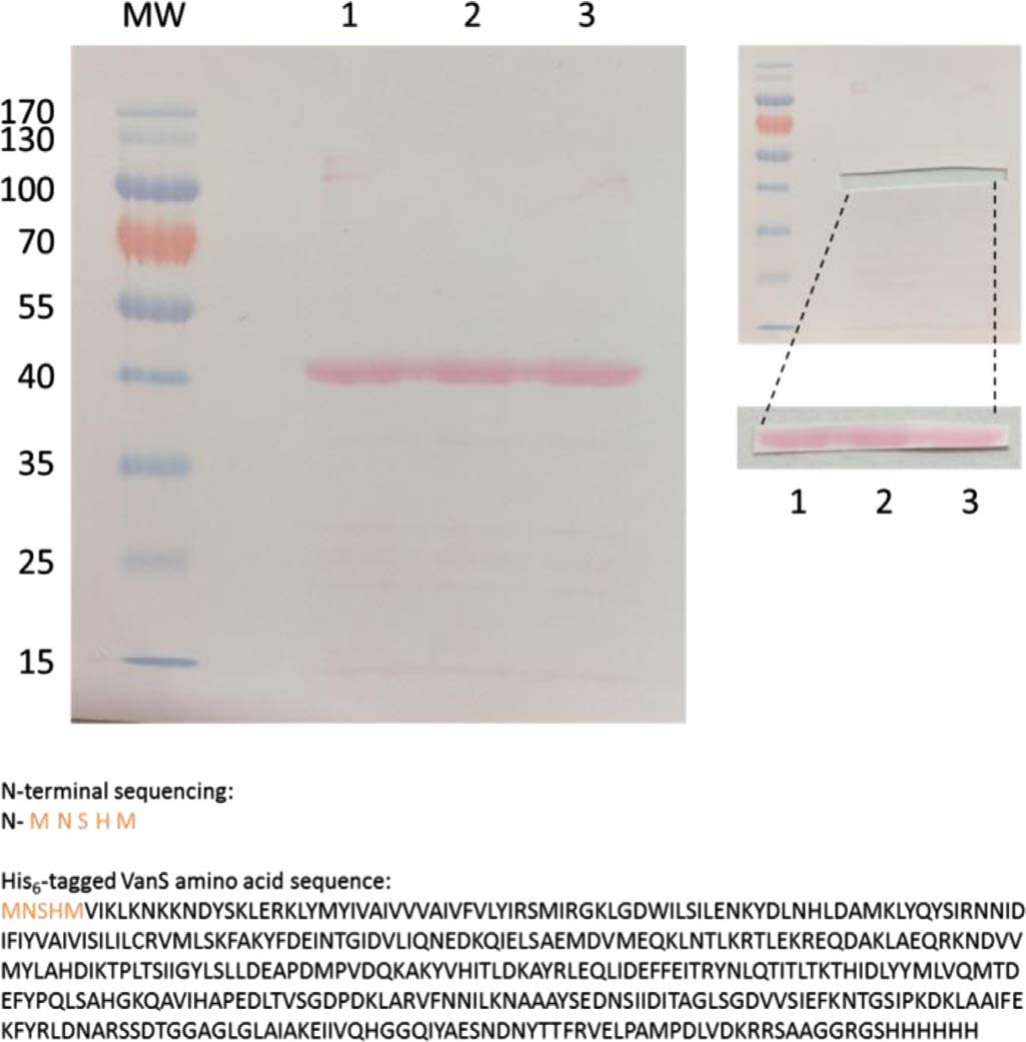
N-terminal sequencing of VanS_A_. Sequence MNSHM confirmed the excised electroblotted purified protein band used for N-terminal sequencing as VanS_A_.

**Fig. 2:**
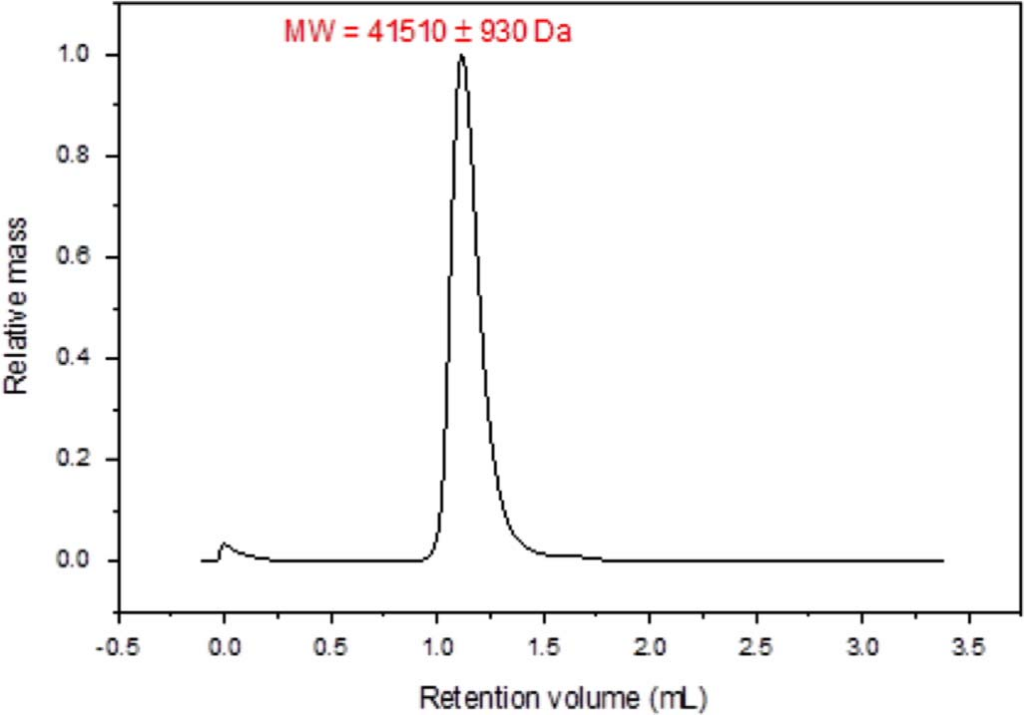
Spectrum of SEC-MALS datum for VanS_A_. A Superdex 200 Increase 5/150 GL column (GE Healthcare) was pre-equilibrated with 10 mM HEPES pH 8.0, 5% glycerol, 0.025% DDM before injection of 200 µl of VanS_A_ (0.5 mg/ml) in 10 mM HEPES pH 8.0, 20% glycerol, 0.025% DDM using an ÄKTA pure system (GE Healthcare). Baseline set and the molecular weight calculated using data processing software (UNICORN 6, GE Healthcare). BSA as reference material was used to calibrate the instrument.

**Fig. 3:**
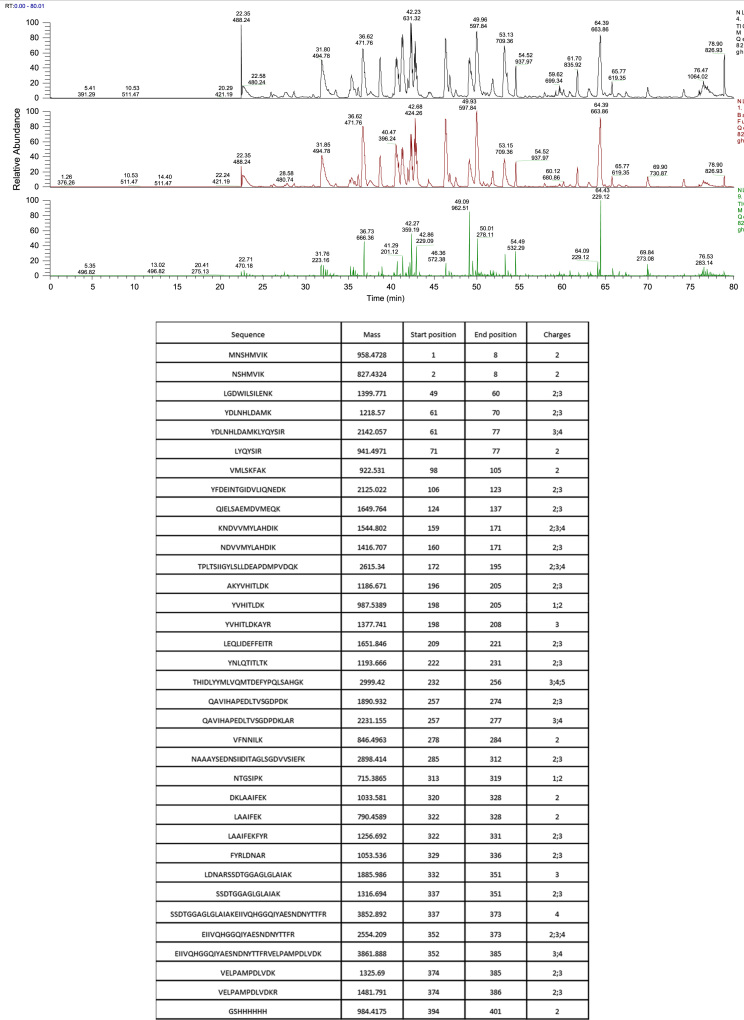
Mass spectrometry results for VanS_A_. LC-MS/MS mass spectrometry of VanS_A_ performed at the Advanced Proteomics Facility, Department of Biochemistry, University of Oxford as described in Methods. (A) Mass spectrometry spectrum for tryspin-digest fragments of VanS_A_; (B) Table of fragment sequences identified using LC-MS/MS showing 35 unique peptides corresponding to the VanS_A_ sequence with 78% of unique sequence coverage giving rise to the molecular weight of the VanS_A_ 45.764 kDa.

**Fig. 4:**
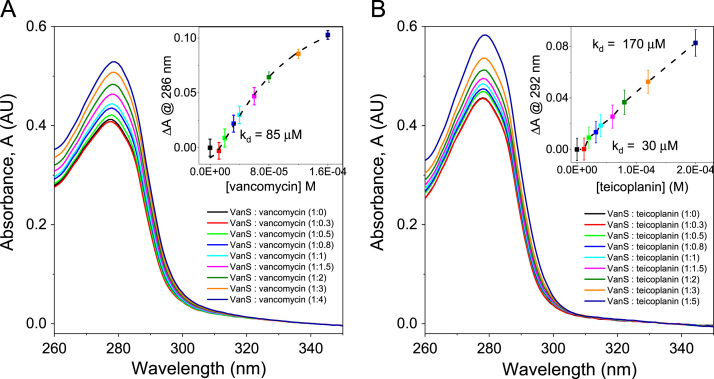
UV absorbance spectrum for VanS_A_ during titrations with (A) vancomycin and (B) teicoplanin. Binding constant (k_d_) calculated from change in Absorbance (AU) at (A) 286  nm and (B) 292  nm, plotting against respective concentration (M) of antibiotic and fitting with a (A) Hill1 or (B) BiDoseResponse function. Standard deviation (*n*=4) shown by error bars.

**Fig. 5:**
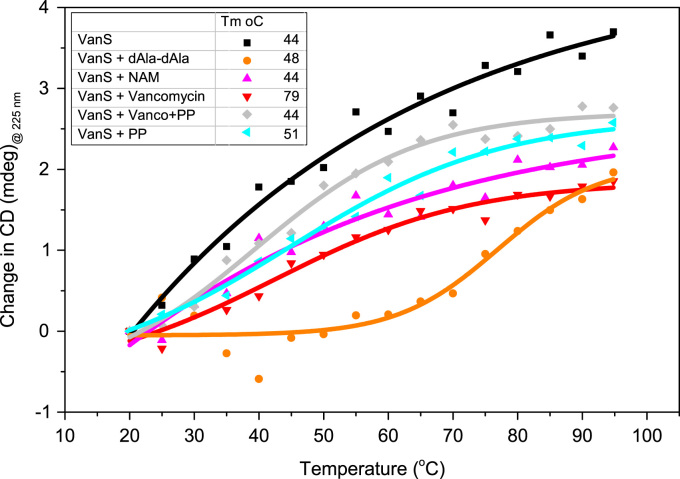
Thermal denaturation profile of VanS_A_ in the presence of ligands (vancomycin, D-Ala-D-Ala, pentapeptide (PP), NAM, PP and vancomycin, and teicoplanin). Change in CD (mdeg) at 225 nm plotted against temperature (°C) and fitted with Gibbs-Helmholtz equation.

Quality control measures for VanS_A_ expression and purification included N-terminal sequencing, Mass Spectrometry and SEC-MALS. Protein of 41,510±930 Da (Fig. 2) and N-terminal sequence MNSHM (Fig. 1) was identified. 78% of the amino acid sequence for VanS_A_ was identified by Mass Spectrometry to give a molecular weight of 45764 Da (Fig. 3). UV absorption measurements during CD titration experiments for VanS_A_ as described in [1] (Fig. 4) were used for quality control of the experimentally determined dissociation constant (k_d_). Change in CD at 225 nm was plotted against temperature and fitted with a Gibbs-Helmholtz equation for determination of melting temperature (T_m_) (Fig. 5).

## 2 Experimental design, materials and methods

### 2.1 N-terminal sequencing

Purified proteins were separated by SDS-PAGE and electroblotted onto PVDF membrane before staining using Ponceau S for visualisation of the bands to be excised for sequencing by Edman degradation at Alta Bioscience Ltd, University of Birmingham, UK.

### 2.2 Size-exclusion chromatography multi-angle light scattering

Performed using 0.1 mg of purified protein in final suspension buffer. A Superdex 200 Increase 5/150 GL column (GE Healthcare Life Sciences) was pre-equilibrated with ddH_2_O overnight, followed by equilibration with running buffer containing 10 mM HEPES pH 8.0, 5% glycerol, 0.025% DDM before sample injection. A flow rate of 0.3 ml/min was used throughout.

### 2.3 Mass spectrometry

LC-MS/MS performed at Advanced Proteomics Facility, Department of Biochemistry, University of Oxford.

Peptides re-suspended in 10% formic acid were separated on an Ultimate 3000 UHPLC system (Thermo Fischer Scientific) and electrosprayed directly into a QExactive mass spectrometer (Thermo Fischer Scientific) through an EASY-Spray nano-electrospray ion source (Thermo Fischer Scientific). The peptides were trapped on a C18 PepMap100 pre-column (300 µm×5 mm,100 Å, Thermo Fisher Scientific) using solvent A (0.1% Formic Acid in water) at a pressure of 500 bar. Peptides were separated on a PepMapRSLC C18 column (2 um, 100 Å, 75 um×50 cm, Thermo Fisher Scientific) using a linear gradient (length: 120 minutes, 7% to 28% solvent B (0.1% formic acid in acetonitrile), flow rate: 200 nL/min). The raw data was acquired on the mass spectrometer in a data-dependent mode (DDA). Full scan MS spectra were acquired in the Orbitrap (scan range 350–2000 m/z, resolution 70000, AGC target 3e6, maximum injection time 50 ms). After the MS scans, the 20 most intense peaks were selected for HCD fragmentation at 30% of normalised collision energy. HCD spectra were also acquired in the Orbitrap (resolution 17500, AGC target 5e4, maximum injection time 120 ms) with first fixed mass at 180 m/z.

Raw MS data were processed by MaxQuant (version 1.5.0.35i) for peak detection and quantification. MS spectra were searched against a custom database using the Andromeda search engine with the following search parameters: full tryptic specifity, allowing two missed cleavage sites, fixed modification was set to carbamidomethyl (C) and the variable modification to acetylation (protein N-terminus), oxidation (M).

Mass spectra were recalibrated within MaxQuant with a precursor error tolerance of 50 ppm and then re-searched with a mass tolerance of 5 ppm.

Fragment ion tolerance was set to 20 ppm.

### 2.4 Synchrotron Radiation Circular Dichroism (SRCD), Circular Dichroism (CD) and UV–vis absorbance

SRCD spectroscopy was carried out in a nitrogen-flushed chamber at beamline B23 at the Diamond Light Source Ltd, Oxfordshire as described in [5], [6]. For CD studies and UV–vis absorbance, experiments were conducted using a Chirascan-Plus (Applied Photophysics).

Ligand-containing samples were performed by addition of 5-fold molar equivalent of ligand stocks in 10 mM Tris. HCl pH 8.0 (control incubated with equivalent volume of 10 mM Tris–HCl pH 8.0). All samples were incubated at 20 °C for 30 min prior to data collection. All data was analysed using CDApps [2] where the mean residue weight of VanS_A_ was taken to be 113. Unless otherwise stated, all spectra presented are difference spectra where all relevant background buffers, ligands etc. have been subtracted. Data acquired when the HT of the detector (PMT) was equal to or greater than 600 V were excluded from the analyses. Far-UV measurements (180–260 nm) were commonly collected using 0.5 mg/ml of VanS_A_ with bandwidth of 1 nm and 1 s integration time. Data presented in molar extinction (Δε). Temperature denaturation measurements were collected over the temperatures (20–95 °C, in 5 °C increments) in the absence and presence of ligands. Samples were incubated at the initial 20 °C for 30 min after ligand or solvent addition prior to data acquisition. At each temperature step, reactions were incubated for 2 min prior to data collection (1 scan). A final scan was acquired post-temperature ramp after returning to 20 °C and incubation for 20 minutes before data acquisition. Data was analysed using CDApps [1] to obtain difference spectra where all controls (buffers, ligands, etc.) had been subtracted. Change in CD (mdeg) at a specific wavelength was transferred to OriginPro® 9 and plotted against the corresponding temperature for fitting using Gibbs-Helmholtz equation derived from Boltzmann distribution [2], [3] sigmoidal two-state denaturation curve to a Boltzmann distribution and the expression modified to include parameters for fitting of thermal denaturation data for the calculation of the melting temperature (T_m_).

Near-UV measurements (260–350 nm) were collected using 1 mg/ml of protein using 10 mm pathlength cell, 2 nm bandwidth, 1 nm increments and 1 s integration. Titration experiments were conducted as described for standard near-UV measurements, with the modification of measurements collected after the addition of incremental volumes of ligand stock as described previously [7], [8]. Change in CD (mdeg) a specific wavelength was monitored, the values transferred to OriginPro® and plotted against respective ligand concentration (M) and fitted with a hill1 binding [9] or biphasic dose response [10] function to determine the k_d_ for binding.
